# Preparation and Characterization of a Novel Multiparticulate Dosage Form Carrying Budesonide-Loaded Chitosan Nanoparticles to Enhance the Efficiency of Pellets in the Colon

**DOI:** 10.3390/pharmaceutics15010069

**Published:** 2022-12-26

**Authors:** Fatemeh Soltani, Hossein Kamali, Abbas Akhgari, Mahboobeh Ghasemzadeh Rahbardar, Hadi Afrasiabi Garekani, Ali Nokhodchi, Fatemeh Sadeghi

**Affiliations:** 1 Department of Pharmaceutics, School of Pharmacy, Mashhad University of Medical Sciences, Mashhad 9177899191, Iran; 2 Targeted Drug Delivery Research Center, Pharmaceutical Technology Institute, Mashhad University of Medical Sciences, Mashhad 9177899191, Iran; 3 Pharmaceutical Research Center, Pharmaceutical Technology Institute, Mashhad University of Medical Sciences, Mashhad 9177899191, Iran; 4 Lupin Pharmaceutical Research Center, Coral Springs, FL 33065, USA; 5 School of Life Sciences, University of Sussex, Brighton BN1 9RH, UK

**Keywords:** budesonide, nanoparticles, chitosan, colon delivery, eudragit, pellets

## Abstract

An attempt was made to conquer the limitation of orally administered nanoparticles for the delivery of budesonide to the colon. The ionic gelation technique was used to load budesonide on chitosan nanoparticles. The nanoparticles were investigated in terms of size, zeta potential, encapsulation efficiency, shape and drug release. Then, nanoparticles were pelletized using the extrusion–spheronization method and were investigated for their size, mechanical properties, and drug release. Pellets were subsequently coated with a polymeric solution composed of two enteric (eudragit L and S) and time-dependent polymers (eudragit RS) for colon-specific delivery. All formulations were examined for their anti-inflammatory effect in rats with induced colitis and the relapse of the colitis after discontinuation of treatment was also followed. The size of nanoparticles ranged between 288 ± 7.5 and 566 ± 7.7 nm and zeta potential verified their positive charged surface. The drug release from nanoparticles showed an initial burst release followed by a continuous release. Pelletized nanoparticles showed proper mechanical properties and faster drug release in acidic pH compared with alkaline pH. It was interesting to note that pelletized budesonide nanoparticles released the drug throughout the GIT in a sustained fashion, and had long-lasting anti-inflammatory effects while rapid relapse was observed for those treated with conventional budesonide pellets. It seems that there is a synergistic effect of nanoformulation of budesonide and the encapsulation of pelletized nanoparticles in a proper coating system for colon delivery that could result in a significant and long-lasting anti-inflammatory effect.

## 1. Introduction

Targeting drug delivery is advantageous for the treatment of inflammatory bowel disease (IBD), including Crohn’s disease and ulcerative colitis (UC) [1,2,3]. An appropriate drug delivery system could allow delivery of the sufficient amounts of drugs with appropriate release rates to the site of its action [4]. Budesonide (BU) is a topical anti-inflammatory steroid, which has proven to be effective in the treatment of IBD [5]. Therefore, targeting this drug at the site of inflammation is an interesting perspective for clinical applications.

Nanoparticles have shown an encouraging and promising outlook for drug or gene delivery in IBD treatment [6,7,8]. The design of drug-loaded nanoparticles for delivery to the colon via oral administration has been used as a strategy to further amplify drug uptake into the inflamed tissue of the colon [1,9]. Nanoparticles have a great chance to reach and accumulate in inflamed tissues due to the loosening of tight junctional complexes [10]. A study on the rat intestinal loop model demonstrated that nanoparticles in the size range of 100 nm allow better penetration in the submucosal layers compared with those in the size range of 500 nm which mainly showed localized targeting to the epithelial lining [11]. Additionally, it has been reported that nano-sized particles showed favored uptake by immune cells whose numbers are high in inflamed tissue [12].

In some studies, triggering drug release into the colon was reported by encapsulation of prednisolone in nanocarriers [13]. Additionally, 5-aminosalicylic acid nanoparticles demonstrated a significant improvement in targeting inflamed tissue in a mouse with UC [14]. Many studies demonstrated that budesonide-loaded nanoparticles have shown promising results for targeted delivery to intestinal mucosa with inflammation [15,16,17].

Limitations such as burst drug release, premature nanoparticle uptake or lack of pH sensitivity have been noticed as obstacles to the efficient transport of drugs to the colon upon oral administration of nanoparticles [4]. For example, the orally administered tacrolimus-nanoparticles showed minor therapeutic effects due to the slow rate of drug release, degradation in an enzymatic environment of the upper parts of the GI tract, or uptake into the systemic circulation followed by hepatic metabolism [1,12].

Based on this consideration, additional strategies such as surface charge-dependent nanoparticles [18], PEGylation-dependent nanoparticles [19], pH-dependent nanoparticles [1,20], hydrogel-based targeting [21], ligand-receptor-mediated targeting [22] and reactive oxygen species [23] are being explored to enhance drug delivery to the areas of inflammation and achieve maximal retention time in diseased tissues for orally administered nanoparticles. Amongst the methods mentioned above, the design of a pH-sensitive nano-delivery system is one of the simplest [24] and most commonly used approaches for the selective delivery of nanoparticles to the site of inflammation [6,25,26]. Many studies demonstrated that pH-dependent BU nanoparticles could alleviate the colitis better than plain nanoparticles [27,28,29]. 

Despite the promising results observed for pH-dependent nanoparticles in targeting the colon, some concerns including the inter- and intra-individual discrepancies in pH in GIT and the disease-related variations in luminal pH could be obstacles to the successful performance of these systems. Encapsulation of nanoparticles in dual pH and time-dependent polymers has been applied as a strategy to decrease initial drug release and activity in the upper parts of GIT in comparison with plain nanoparticles [29]. While such systems demonstrated better therapeutic effects than single pH or time-dependent systems, premature drug absorption in the upper region of GI due to their small size should also be considered [30]. It should also be mentioned that the systems described to date such as entrapped nanoparticles in enteric microparticles require two steps of emulsification and solvent evaporation techniques for preparation [31,32]. On the other hand, the use of organic solvents for solubilizing pH-sensitive polymers could probably increase the leaching of the drug and result in a decrease in the drug content of the nanoparticles [33], and a lack of reproducibility [34]. To overcome these limitations researchers embedded BU lipid-based nanoparticles in enteric-coated pellets to reach the exact site of action [30].

Since lipid-based nanoparticles might be unstable in the GI tract [35], and also due to the superiority of polymeric to lipid-based formulations of BU nanoparticles in the treatment of IBD [36], polymer-based nanocarriers for specific delivery of BU to the colon would be desirable.

Chitosan (CS) as a cationic natural polysaccharide with desirable biodegradability, biocompatibility and mucoadhesive properties [16] has been extensively used in colon targeting delivery systems [37,38,39]. Chitosan-based nanoparticles could adhere to the surface of intestinal mucosa in inflamed tissues via the interaction between the positively charged nanoparticles and the negatively charged intestinal mucosa [40] and for drugs such as BU with local action this perspective might be beneficial.

In this study, to overcome the limitations of orally administered nanoparticles for delivery of BU to the colon, an attempt was made to design a coated multiparticulate dosage form (pellets) carrying budesonide-loaded chitosan nanoparticles. The coating was composed of combined pH and time-dependent eudragits to minimize the early drug release and absorption in the upper sections of the GI tract and maximize delivery of the drug-loaded nanoparticles to the colon. 

## 2. Materials and Methods

### 2.1. Materials

Budesonide (BU) was acquired from Jaber Ebne Hayyan Pharmaceutical Company (Tehran, Iran). Lactose monohydrate and Avicel pH 102 were obtained from Merck, Frankfurt, Germany. Polyvinylpyrrolidone (PVP K30) (Rahavard Tamin, Saveh, Iran), eudragit S PO, (Evonik Industries AG, Hanau, Germany), eudragit L PO, (Evonik Industries AG, Hanau, Germany), eudragit RS PO, (Evonik Industries AG, Hanau, Germany), talc, (Merck, Frankfurt, Germany), triethyl citrate (TEC) (Merck, Frankfurt, Germany), chitosan (medium-molecular-weight, 75–85% deacetylated) (Sigma-Aldrich, St. Louis, USA), three polyphosphate (Merk, Frankfurt, Germany), acetic acid (Dr. Mojallaly, Tehran, Iran), ethanol (Dr. Mojallaly, Tehran, Iran), isopropyl alcohol (2-propanol) (Dr. Mojallaly, Tehran, Iran) and sodium lauryl sulfate (SLS) (Scharlau, Barcelona, Spain) were utilized. All other reagents and solvents were of analytical grades.

### 2.2. Preparation of Nanoparticles

Budesonide-loaded chitosan nanoparticles (BCN) were made based on the ionotropic gelation technique [41]. First, 100 mg of chitosan was dispersed and dissolved in 50 mL of deionized water containing 1% *v*/*v* acetic acid (pH 4.7) to obtain the homogeneous solution (2 mg/mL). Second, ethanol was employed to dissolve BU (the minimum volume of ethanol was used). The BU ethanolic solution was added to chitosan solution at a different drug: chitosan ratios (1:10, 2:10, 3:10 and 4:10 *w*/*w*), followed by magnetic stirring at 700 rpm for 30 min (Heidolph Instruments, MR Hei-Tec, Schwabach, Germany). This solution was heated to 60 °C for 10 min. An aqueous solution of three polyphosphate (TPP) (1 mg/mL at 8 °C), was added dropwise to the warm chitosan solution under continuous stirring (700 rpm) at room temperature. The weight ratio of chitosan to TPP in all formulations was kept at 3:1 based on previous studies [42,43]. After the addition of TPP, stirring was continued for 1 more hour at room temperature. The resulting suspension was centrifuged at 21,000 rpm at 4 °C for 30 min (Sigma, 3–30 K, Schnelldorf, Germany) to separate nanoparticles. The nanoparticles were washed with deionized water and then freeze-dried for 48 h (Heto, Dw 3, Allerd, Denmark) using sucrose 1.5% *w*/*v* as lyoprotectant. The freeze-dried nanoparticles were maintained in a closed container for further use. The same procedure was followed for the preparation of drug-free chitosan nanoparticles (CN) for comparison purposes.

### 2.3. Physicochemical Characterization of Nanoparticles

#### 2.3.1. Particle Size Distribution Study

The particle size distribution and zeta potential of nanoparticles were assessed by dynamic light scattering (DLS) (Zetasizer 5000, Malvern, UK). The obtained nanoparticles were diluted 10 times by adding 900 µL of distilled water to 100 µL of the solution containing nanoparticles before measurement. All measurements were carried out in triplicate at room temperature.

#### 2.3.2. Encapsulation Efficiency and Yield

The amount of BU entrapped within the nanoparticles was measured directly by dispersing 20 mg of nanoparticles in a solvent mixture containing 5 mL of acetic acid 1% *v*/*v* (pH 4.7) and 5 mL of ethanol. This suspension was shaken at 50 rpm by a mechanical shaker (TUV-NORD, Tehran, Iran) for 72 h at room temperature, and then filtered via a disposable syringe filter (pore size 0.22 μm). The concentration of BU in the filtrate was examined by a UV spectrophotometer at 246 nm (Shimadzu, UV/1204, Tokyo, Japan). Specimens were prepared in triplicate, and encapsulation efficiency (%) (EE) was analyzed by Equation (1) below:

(1) $$\mathrm{Encapsulation}\;\mathrm{efficiency}\;(\%)\;\frac{Amount\;of\;budesonide\;in\;NPs}{Amount\;of\;budesonide\;initially\;added}\times 100$$

Additionally, the production yield (%) for the selected formulation was calculated using the following equation:

(2) $$\mathrm{Yield}\;(\%)=\frac{Weight\;of\;nanoparticles}{Initial\;weight\;of\;polymer\;and\;drug}\times 100$$

#### 2.3.3. Transmission Electron Microscopy (TEM)

In order to observe the morphology of the selected nanoparticles (nanoparticles with 1:10 drug:chitosan) transmission electron microscopy (TEM) (Jeol JEM-1400, JEOL Ltd.; Tokyo, Japan) was employed. A drop of the aqueous dispersion of the washed nanoparticles was deposited in a mesh copper grid and air-dried at room temperature. Then, the grid was subjected to a 60 kV acceleration voltage.

#### 2.3.4. Scanning Electron Microscopy (SEM) 

To evaluate the external morphology of the selected nanoparticles after freeze-drying scanning electron microscope (Leo, VP1450, Neu-Isenburg, Germany) was employed. Freeze-dried nanoparticles were spread on a black steel grid and then coated with a thin film of gold using a gold sputter (Polaron, SC7620 sputter coater, Laughton, England) for 180 s under argon atmosphere. Voltages of 5 kV were selected for accelerating the electrons from electron gun onto the specimen.

#### 2.3.5. Differential Scanning Calorimetry (DSC)

To evaluate the thermal property of nanoparticles, a differential scanning calorimeter calibrated with indium standard was used (Mettler Toledo, DSC 822e, Greifensee, Switzerland). The samples (3–5 mg) were hermetically sealed in DSC pans and were scanned in the temperature range of 25–300 °C at a rate of 10 °C/min under a nitrogen flow of 80 mL/min.

#### 2.3.6. X-ray Powder Diffraction (XRPD)

XRPD study of nanoparticles with 1:10 drug:chitosan was carried out by employing an X-ray powder diffractometer (GNR, Explorer, Milan, Italy). The instrument was operated at 40 kV and 30 mA in the range (2θ) of 5 to 55° using a step size of 0.01° 2θ and step-time of 3 s, with a non-stop mode.

#### 2.3.7. FTIR

Selected nanoparticles (1:10 drug:chitosan) were analyzed with an FTIR spectrometer (Thermo Nicolet, AVATAR 370, Walthman, USA) from 400 to 4000 cm^−1^ at room temperature by the KBr disc method.

#### 2.3.8. In Vitro Drug Release Studies

The release pattern of BU (powder), as well as the selected BCN (1:10 drug:chitosan) corresponding to 9 mg of BU (*n* = 6) were studied in 250 mL of simulated gastric fluid (SGF) pH 1.2 and simulated colonic fluid (SCF) pH 6.8 comprising 0.25% *w*/*v* SLS. USP dissolution apparatus I (Pharmatest, PTWS 3E, Hainburg, Germany) was employed to study the release pattern of BU from the selected formulations. The dissolution was operated under the rotation speed of 75 rpm, at 37 ± 0.5 °C. In the case of BU powder and freeze-dried BCN, an accurately weighed sample was gently dispersed in the medium. At pre-defined time intervals, samples (5 mL) were withdrawn from the release medium and filtered through a disposable syringe filter (pore size 0.22 μm). Subsequently, the amount of BU in 1 mL of filtrate sample was quantified using HPLC (Shimadzu, Kyoto, Japan) equipped with a Teknokroma column (BRISA LC2 C18 250 mm × 4.6 mm, 5 μm). The mobile phase consisted of an acetate buffer (pH 3.9) and acetonitrile mixture (35:65) flowing at a rate of 1.5 mL/min and BU was measured at 240 nm. 

### 2.4. Pelletization of Budesonide Nanoparticles 

The extrusion–spheronization technique was used to load selected BCN (the formulation with the smallest size and highest encapsulation efficiency) into the pellet formulation. For the preparation of pellets containing budesonide-loaded nanoparticles (BCNP) with 1% *w*/*w* of the drug, a powder blend containing 25% *w*/*w* freeze-dried selected BCN, 2% *w*/*w* PVP K30, 56% *w*/*w* Lactose mono hydrate and 17% *w*/*w* Avicel^®^ PH 102, were mixed by a kitchen mixer (FUMA, Fu-1877 Hand Mixer, Tokyo, Japan) for twenty minutes. The mixture was wetted by adding distilled water. The wet mass was passed through an axial screw extruder (Dorsa Tech, EX-01, Tehran, Iran). The extrusion was performed under the rotation speed of 100 rpm using flat sieves of 1 mm aperture size. Then, the extrudates were spheronized (Dorsa Tech, EX-01, Tehran, Iran) for 5 min using a cross-hatched friction plate rotated at 1200 rpm. The obtained pellets were dried in an oven (24 h at 40 °C) and followed by sifting through 1180 and 850 μm. The sifted pellets between 1180 and 850 μm were collected. 

Conventional BU pellets (CP) were also manufactured according to the method established in our previous study [44]. In brief, all powdered components, including 1.5% *w*/*w* BU, 5% *w*/*w* PVP K30, 68.5% *w*/*w* lactose mono hydrate and 25% *w*/*w* Avicel^®^ PH 102 were mixed for 20 min and then turned to cohesive mass using distilled water. The wet mass was extruded and spheronized according to the methods described for BCNP.

### 2.5. Evaluation of Pellets 

#### 2.5.1. In Vitro Drug Release Studies

The release profiles of uncoated BCNP corresponding to 9 mg of BU (*n* = 6) were studied as described in Section 2.3.8. 

#### 2.5.2. Pellet Morphology Studies

The morphology of BCNP and CP was analyzed by measuring the sphericity and aspect ratio of pellets. Pictures were taken by a stereomicroscope (Kyowa, Tokyo, Japan) equipped with a computer system and video camera (Sony, Tokyo, Japan) and then analyzed by an image analyzing software (ImageJ 1/50 for windows).

#### 2.5.3. Particle Size Analysis of Pellets

The particle size of BCNP and CP was analyzed using the sieve method. A sample of 25 g of pellets was shaken on top of the series of standard sieves (150, 180, 250, 425, 850, 1000, and 1180 μm) using a sieve shaker (Azmun test, 50410, Tehran, Iran) for 10 min. The mass of pellets that remained on each sieve was determined and used to calculate the geometric mean particle size (dg) and geometric standard deviation (σg) from the plot of the cumulative percentage of undersize on the probability scale versus the log of particle diameter.

#### 2.5.4. Pellet Mechanical Properties

The mechanical characteristics of BCNP and CP were examined by testing twenty pellets in the size range of 850–1180 μm using a Material Testing Machine (Hounsfield, H50KS, London, England). Force–displacement graphs were prepared by a computer program connected to the device (Hounsfield, QMAT, London, England). The 1 kN load cell was set up at a moving speed of 1 mm/min to determine the pellet crushing strengths and elastic modulus. 

### 2.6. Coating of Pellets

CP and BCNP were coated using a Wurster column fluid bed coater (Haltingen-Binzen, UNI-Glatt, Binzen, Germany). A solution of 48% *w*/*w* eudragit S, 12% *w*/*w* eudragit L and 40% *w*/*w* eudragit RS was prepared in a mixture of isopropanol and distilled water (9:1) under agitation. Triethyl citrate (TEC) was included as a plasticizer (10% *w*/*w* based on the weight of polymers) and stirred for 1 h. Talc was then added as an antiadhesion agent (5% *w*/*w* based on polymer weight). The coating solution was utilized onto 50 g pellets where the inlet air and outlet air temperatures were adjusted at 43–40 °C and 39–36 °C, respectively. In this experiment, the atomization pressure and spray rate were set up at 2 bar and 0.5 g/min, respectively. The coating process was continued till the coating mass reached 10% (*w*/*w*). 

### 2.7. In Vitro Drug Release Studies of Coated Pellets

The evaluation of the drug release pattern from coated pellets was performed according to the continuous mode of the dissolution test at 37 ± 0.5 °C (USP dissolution apparatus I, Pharmatest, PTWS 3E, Hainburg, Germany). The amounts of weighed pellets (*n* = 6) equal to 9 mg of BU were tested in 250 mL of a medium comprising 0.25% *w*/*v* SLS at a rotation speed of 75 rpm. Dissolution tests were performed at various time intervals of 2 h (pH 1.2), 1 h (pH 6.5), 2 h (6.8), 1 h (pH 7.2) and 10 h (pH 6.8). The dissolution was also performed in the medium containing 4% *w*/*v* rat cecal content with pH 6.8 to simulate GIT media and the transit time of the pellets from various sections (i.e., stomach, duodenum, jejunum, ileum, and colon). The media supplemented by cecum content was continuously bubbled with CO2. After each incubation time for each medium, the baskets with their pellets were moved rapidly from one medium to another. At defined time intervals, samples (5 mL) were withdrawn manually and analyzed by HPLC according to the method explained in Section 2.5.1. In the case of media supplemented by cecum content, 1 mL of the medium was removed and centrifuged at 15,000 rpm at 4 °C for 30 min. Then, the supernatant was taken and diluted with acetonitrile at a ratio of 1:3 and the mixture was filtered using a disposable syringe filter (pore size 0.22 μm) before the sample is injected to the HPLC column [45].

### 2.8. Morphology of Coated Pellets 

The surface morphology as well as cross section of the coated BCNP were characterized using scanning electron microscope (Leo, VP1450, Neu-Isenburg, Germany). The pellets were fixed on an aluminum stub and sputter-coated (Polaron, SC7620 sputter coater, Laughton, England) with a thin layer of gold for 180 s using a sputter coating machine under argon atmosphere, and then analyzed using SEM. Voltages of 5 kV were selected for accelerating the electrons from electron gun onto the specimen. The cross-sectional samples were prepared using a razor. These samples were then covered with a thin layer of gold.

### 2.9. Animal Treatment

To investigate the efficacy of different formulations of BU in the treatment of colonic damage, the rat model of ulcerative colitis was employed. Wistar male rats aged 10–12 weeks and weighing 250–300 g were used in this part of the study, under institutional guidelines granted by the institutional ethics committee of Mashhad University of Medical Sciences (IR.MUMS.REC.1399.011).

All animals employed in the study were kept in a clean room, with air-conditioned (22 ± 3 °C), controlled humidity (40 ± 5%) and light–dark cycles of 12 h; 24 h before the colitis induction, all the rats were weighed, examined to be healthy and kept fasted except for water. For induction of colitis, first of all, an intraperitoneal injection of ketamine (50 mg/kg) and xylazine (5 mg/kg) solution was used to anaesthetize rats [46,47]. Thereafter, acetic acid (2 mL 3% *v*/*v* in normal saline) was administered rectally 8 cm deep using a 2 mm diameter cannula [48]. The healthy control group received 2 mL of normal saline solution intrarectally. Rats were kept in an upside-down position for 1 min and then they were taken back to their cages. All animals were left for 3 days without treatment to let the colitis to be developed while they were free for consumption of water and food [49]. The colitis-induced rats were then divided randomly into 7 groups, with 6 animals per group. One group of rats were used as the control group (untreated group), whilst others were treated with conventional coated pellets (coated CP), pellets containing BU loaded nanoparticles (BCNP), coated BCNP, BU nanoparticle (BCN), free drug chitosan nanoparticles (CN) and free BU powder (BU) three days after induction of colitis. Each treated group received orally an equal dose of BU (0.15 mg/day) for 7 consecutive days [50]. In the case of BCN, CN and BU, the dose was suspended in 1 mL of purified water before administration. The healthy and the untreated control groups received 1 mL of normal saline. The weight and stool consistency of the rats were evaluated during this period. The rats were killed by CO_2_ asphyxiation either 24 h or 6 days after the last dose administration. Then, the colon, from the cecum to the anus was cut for further studies. 

### 2.10. Evaluation of Colitis Treatment 

#### 2.10.1. Colitis Activity Index

The degree of inflammation was checked daily by determining the colitis activity index (CAI) which consists of three clinical parameters including weight loss, stool consistency, and anal bleeding [51]. Briefly, the score scale from 0 to 4 indicates the degree of inflammation (from healthy to maximal inflammation). Scores of 0, 1, 2, 3 and 4 correspond to no weight loss, 1–5% weight loss, 5–10% weight loss, 10–20% weight loss and more than 20% weight loss, respectively. Similarly, for consistency of stool, a well-formed pellet gets 0, pasty and semi-formed stools with no sign of stickiness to the animal’s anus gets 2, and liquid stools with stickiness to the anus gets 4. In addition, bleeding scores were 0 for no blood, 2 for positive findings, and 4 for gross anal bleeding. The average of these points was reported as CAI. 

#### 2.10.2. Colon/Body Weight Ratio

To determine the colon/body weight ratio, rats were weighed before scarification. Colon tissue samples were resected, cut longitudinally, and washed with an iced phosphate-buffered solution (pH 7.4). The weight of washed resected colon was also determined and the ratio of the wet colon weight to the body weight of each rat was defined as an index of colonic inflammation [31]. 

#### 2.10.3. Weight/Length Ratio of Colon

The length and wet weight of the washed colon tissue samples was assessed, and the weight/length ratio was determined as a sensitive and reliable indicator of the severity of the colonic inflammation [52].

#### 2.10.4. Glutathione Content of the Colon Tissue 

The amount of reduced glutathione in the colon tissue was defined spectrophotometrically based on the appearance of yellow color after the addition of DTNB (5,5’-dithiobis-(2-nitrobenzoic acid)) to compounds bearing sulfhydryl groups [53]. In brief, 0.5 mL of tissue homogenate 10% *w*/*v* in phosphate-buffered saline (0.1 M, pH 7.4) was blended with 0.5 mL of 10% *w*/*v* trichloro acetic acid (TCA) and vortexed. The blend was then centrifuged at 2500× *g* for 10 min at 4 °C and 0.5 mL of supernatants was withdrawn and mixed with reaction mixtures consisting of 2.5 mL phosphate buffer (pH 8) and 0.5 mL DTNB. The absorbance of the mixture was determined at 412 nm spectrophotometrically (Jenway 6105 UV/Vis, Staffordshire, UK) and GSH contents (nmol/g tissue) were assayed against GSH standard curve prepared in the blank medium [54].

#### 2.10.5. Malondialdehyde Content of the Colon Tissue 

The malondialdehyde (MDA) concentration of the colon tissue which is related to the severity of colitis was measured [55]. Briefly, colon specimens were frozen in liquid N_2_ rapidly after scarification and stored for subsequent evaluation. To determine MDA concentration (nmol/g tissue), 3 mL phosphoric acid (1% *w*/*v*) and 1 mL thiobarbituric acid (TBA) (0.6% *w*/*v*) were introduced to colon tissue homogenate (10% *w*/*v*) in KCl (1.15% *w/v*). The mixtures were heated in a water bath for 45 min. Then, 4 mL of n-butanol was added to the cooled mixture and vortexed for 1 min. The mixture was then centrifuged at 3000× *g* for 10 min. Then, absorbance of the supernatant at 532 nm was determined by a spectrophotometer (Jenway 6105 UV/Vis, Staffordshire, UK) and the MDA concentration was calculated based on the constructed standard curve obtained in the blank medium [56].

#### 2.10.6. Histological Assessment of Colitis Severity

A segment of the washed colon tissue specimens was fixed in a 10% formalin solution and inserted in paraffin. Cutting sections obtained by microtome were stained with hematoxylin and eosin (H&E) and examined by optical microscope to assess the severity of colitis. The colitis severity was scored from 0 to 4 based on the microscopic observation of cross-sections of the colon. The severity of colitis was scored 0 when no or minor inflammation was observed. When there was a trace region of focal inflammatory cell infiltration, the severity of colitis was scored 1. When a major part of the colon tissue was exposed to inflammation and sign of smooth muscle thickening was observed, the severity of colitis was scored 2. If ulcerated regions and inflammatory cell infiltration were formed in the tissue sections following the inflammation, the severity of colitis was assigned a score of 3. Finally, the highest colitis severity score (i.e., 4) was assigned when the entire tissue damage appeared as necrosis and gangrene [45].

#### 2.10.7. Blood Glucose Level

The blood glucose level in rats was measured from the 12th day until the 17th day of the study by sampling the tail vein blood with an 18-gauge needle in the strip of a blood glucose meter (Accu-chek^®^ Active, Roche, Mannheim, Germany). This experiment could provide some comparison amongst specific colon delivery potential of different formulations investigated [57,58], and also information regarding the potency of nanoparticles for accumulation in inflamed regions. Data on blood glucose levels are stated in mg/dL and compared with untreated animals [48].

### 2.11. Statistical Analysis

To analyze the obtained data statistically, Graph Pad Prism software (Graph Pad Prism, version 7, San Diego, CA, USA) was employed. One and two-way analyses of variance (ANOVA) followed by Tukey–Kramer test was employed to compare the differences between means (*p* < 0.05 is an indication of a significant difference).

## 3. Results and Discussion

### 3.1. Preparation and Characterization of Nanoparticles

The anti-inflammatory effect of chitosan against IBD and UC has been reported in previous studies [59] where high-molecular-weight chitosan had better therapeutic effects in UC compared with-low molecular-weight chitosan [60]. On the other hand, an increase in the molecular weight of chitosan could result in a decline in the drug release which could be due to the greater viscosity of the gel layer created around the drug particles upon contact with the dissolution medium [61,62]. Since BU is a highly hydrophobic drug with low water solubility [63], its slow release could provide an insufficient concentration of the drug at the site of action. Furthermore, it has been reported that in the ionic gelation procedure for producing chitosan nanoparticles, medium-molecular-weight chitosan has shown more encapsulation efficacy compared with high-molecular-weight chitosan [64]. Therefore, medium-molecular-weight chitosan had been used in this study in an attempt to prepare BU nanoparticles by the ionic gelation technique [41,42]. This method has great potential for industrial application due to the lack of need for use of organic solvents, sonication or high temperatures and also the relative easiness of scaling up [65]. The production of nanoparticles with a lower polymer content could lead to a more cost-effective formulation; therefore, in the current study, the effect of the drug-to-chitosan ratio in the preparation of nanoparticles was investigated to find out if it is possible to achieve desirable properties for nanoparticles at low concentrations of chitosan in formulations. 

The size characteristics of nanoparticles have been found to affect drug accumulation in the inflamed colon [66]. Despite the fact that larger size nanoparticles (500 nm) are mainly localized in the epithelial lining [11,67], it has been demonstrated that excellent mucoadhesive properties belong to the nanoparticles with a particle size range between 200 and 300 nm [68]. In this study, the particle size of the different formulations ranged between 288 ± 7.5 and 566 ± 7.7 nm with small PDI values indicating the production of monodispersed particles. Table 1 shows that with an enhancement in the ratio of drug: chitosan (from 1:10 to 4:10), larger nanoparticles with lower encapsulation efficacy (37% to 2%, respectively) were obtained. The nanoparticles are formed through inter and intramolecular interactions between the negative groups of TPP and the positive charged amino groups of chitosan [69]. It has been found that chitosan molecules have a spread conformation in solution due to the electrostatic repulsion forces between free amino groups along the molecular chain [70]. These free amino groups are also responsible for the electrostatic interactions between the nanoparticles, helping to reduce particle sizes [70]. Upon the addition of the BU to the chitosan solution under constant stirring, oxygen atoms of BU interact non-covalently with the free amino groups of chitosan chains [41], leading to a decrease in the number of free amino groups. Since the concentration of free amino groups can have an impact on the creation of nanoparticles [62] and the encapsulation efficiency depends on the number of available sites present in the formulating material [70], the higher concentration of BU resulted in the formation of larger nanoparticles as well as lower encapsulation efficacy. Similar results have been reported in other studies [41,70,71].

All prepared formulations showed positive zeta-potential ranges from +26.6 ± 1.1 to +31.0 ± 1.2 mV. Since BU is a slightly anionic molecule [72], positive zeta potentials are due to the positively charged amino groups of chitosan molecules present at the surface of particles. It can be postulated that nanoparticles with zeta potential below −25 mV and above +25 mV can be considered stable at physiological pH [73]; therefore, all nanoparticles produced in the current study can be expected to be stable under physiological conditions.

According to data shown in Table 1, the formulation with a drug:chitosan ratio of 1:10 (BCN-1) provided the smallest particle size and highest encapsulation efficiency (37.2%). Thus, this formulation was chosen, as a representative candidate, for additional studies and was assigned as BCN in the next paragraphs. The yield of the production process of this formulation was 51.0 ± 1.2% which was considered satisfying, taking into account the initial low amounts of the processed material. 

**Figure 1:**
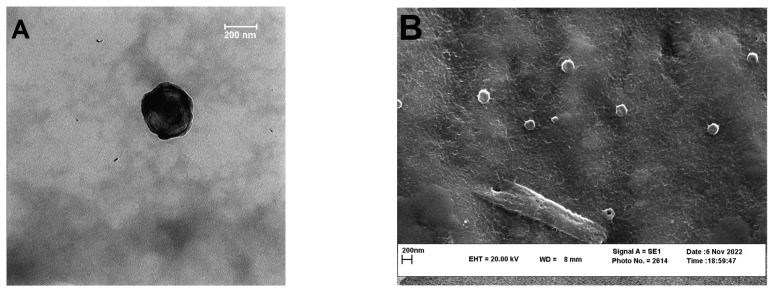
(**A**) TEM image of BCN; and (**B**) SEM images of the freeze-dried BCN.

The morphology of the selected nanoparticle (BCN), as well as freeze-dried BCN, was observed by TEM and SEM, respectively. As it can be seen in Figure 1A,B, the nanoparticles were spherical with almost smooth surfaces. The microscopic images of BCN nanoparticles demonstrated a smaller average diameter in comparison with the DLS results. Such this difference which was also observed in other studies [16,74,75], could be attributed to the fact that in DLS the hydrodynamic diameter of the nanoparticles which is a combination of the diameter of nanoparticles plus the solvent layer attached to them [75] are measured, while in the microscopic image the hydration layer is not present [76]. The absence of major changes in nanoparticle diameter before and after freeze drying (TEM images in comparisons with SEM images) could be related to the proper concentration and good functioning of the lyoprotectant used [77].

**Figure 2:**
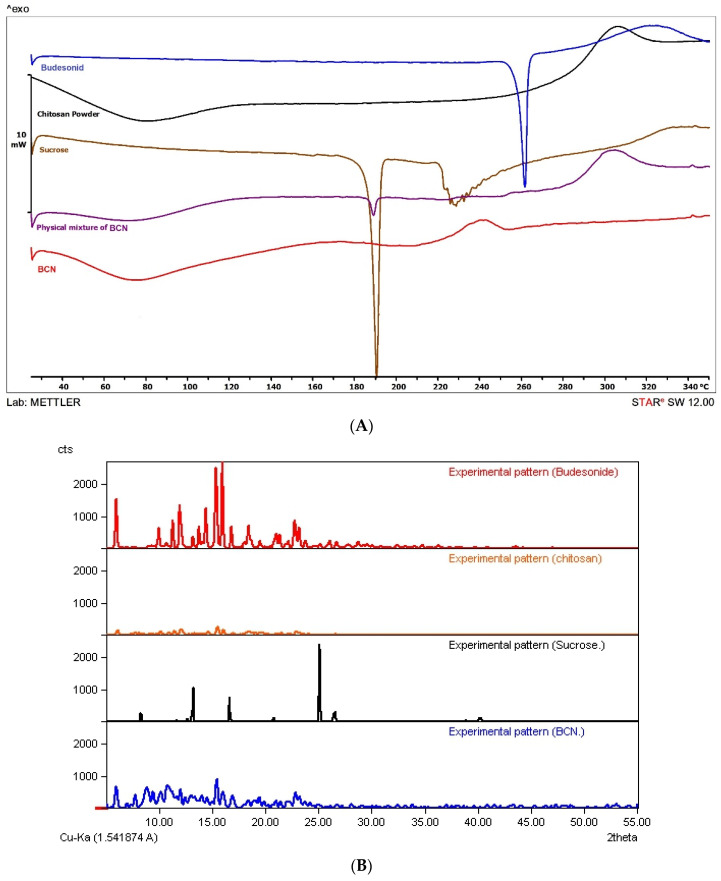
(**A**) DSC thermograms and (**B**) XRPD spectra of budesonide, chitosan, sucrose and BCN.

Figure 2A shows thermograms of the drug powder as well as the chitosan, sucrose, BCN and physical mixture of the excipients used in the preparation of BCN matrix. Budesonide and sucrose are crystalline compounds. Budesonide, as stated by the literature, demonstrated a sharp endothermic melting peak at around 261 °C [41], and sucrose showed an endothermic melting peak at around 190 °C [78]. In contrast, chitosan showed a broad endothermic peak at 100 °C (corresponding to the loss of water) and an exothermic peak at around 310 °C (related to the decomposition of amine units) [79]. The peak related to the melting of sucrose as well as the decomposition peak of the chitosan could be observed in the thermogram of the physical mixture, while the chitosan decomposition peak shifted towards lower temperatures in the BCN thermogram. This shift could be ascribed to the rearrangement of an intermolecular and intramolecular network of the chitosan, due to the crosslinking with the TPP ions as reported elsewhere [41] and also probable interaction between chitosan and the drug. The characteristic peaks of BU disappeared in both BCN and physical mixture thermograms probably either due to the very low content of the drug or presenting in the molecularly dispersed state [44,57]. 

Figure 2B shows the XRPD spectra of the drug as well as the chitosan, sucrose and BCN. Chitosan is a semi-crystalline polymeric material with two characteristic peaks at 2θ of 11 and 21 [80]. The characteristic peaks of BU appeared at 2θ of 5.99, 11.95, 14.42, 15.36 and 15.96 which confirmed the crystalline nature of the drug have also been reported elsewhere [28]. The XRPD spectra of BCN exhibited the characteristic peak of BU with reduced intensity confirming the reduced crystallinity of the drug in nanoparticles.

**Figure 3:**
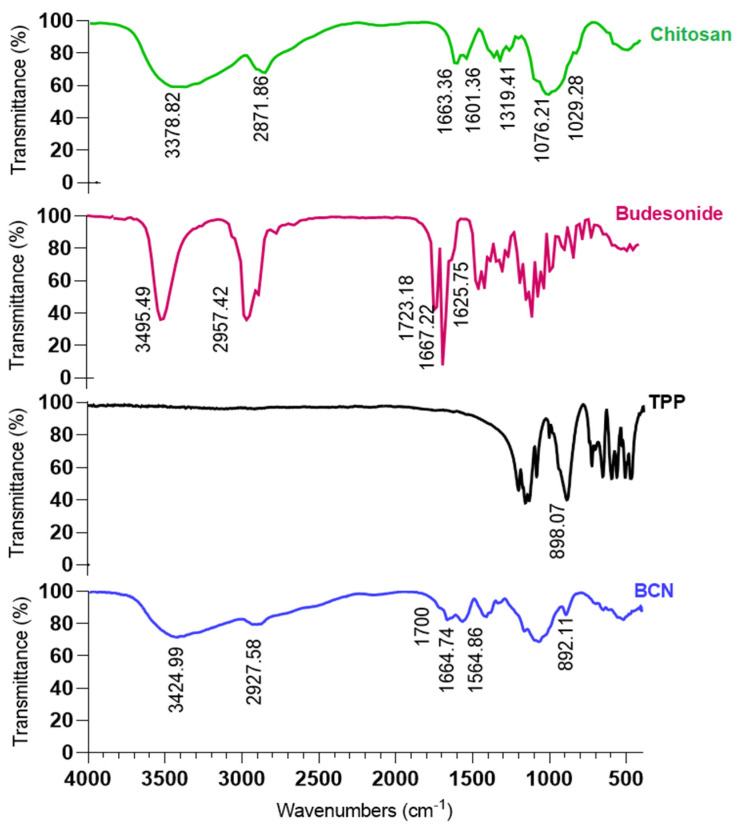
FTIR spectra of chitosan, budesonide, TPP and BCN.

FTIR analysis was performed to characterize the interactions between chitosan and TPP as well as possible interaction between chitosan and BU in nanoparticles (Figure 3). The budesonide spectrum presented peaks at 3495 and 2957 cm^−1^ that correspond to the OH groups and CH2 groups, respectively. Additionally, at 1723 and 1667 cm^−1^ the characteristic stretching peaks of C=O groups as well as stretching peaks of the double bond C=C at 1625 cm^−1^ could be seen [81]. Chitosan showed absorption bands for O-H and N-H2 stretching at 3378 cm^−1^, C-H stretching at 2871 cm^−1^, amides and primary amines at 1663 and 1601 cm^−1^, respectively, C-N bond at 1319 cm^−1^ and ultimately C-O bonds at 1076 and 1029 [41]. The characteristic absorbance peaks of the TPP, related to the phosphate groups presented at 898 cm^−1^ [82], were shifted to lower values (892 cm^−1^) in BCN spectra. This shift could result from interactions between amino groups of chitosan and the phosphate groups of TPP [82]. Almost all characteristic peaks of the BU shifted to the lower values in the FTIR spectrum of the BCN which could account for the interaction between BU and the amino or hydroxyl groups of chitosan. Furthermore, in the BCN spectrum, a new peak at 1565.4 cm^−1^ could be seen that was neither observed in BU nor in chitosan spectrum. This new peak could be related to the N-H2 stretching bond and confirms the presence of hydrogen bonds between BU and chitosan in BCN and could be considered as evidence of the loading of BU into the chitosan nanoparticles.

### 3.2. Preparation and Characterization of Pellets 

The freeze-dried BCN was loaded into the pellets in order to improve the local stability within the gastrointestinal tract and prevent premature uptake of nanoparticles. The extrusion–spheronization technique is a promising technology for converting nanoparticles into solid dosage forms [83]. Conventional pellets of BU were also made for comparison purposes. The formulation of conventional pellets was developed in our previous study [44].

Pellets containing nanoparticles (BCNP) were nearly spherical and had acceptable roundness with the mean sphericity and aspect ratios of 0.98 ± 0.06 and 1.14 ± 0.07, respectively [84]. The geometric mean diameter and standard deviation of BCNP were 1082.1 ± 1.3 μm which indicates the suitability of these formulations for consideration as multiparticulate dosage forms [85]. Additionally, the results of crushing strength (6.3 ± 0.9 N) and elastic modulus (1447.0 ± 170.2 MPa) of BCNP showed that these pellets had suitable mechanical properties for coating steps [86].

The pellets were subsequently coated with combined pH and time-dependent eudragits to prepare a colonic drug delivery system with a sustained release of the drug. Since the pH variations in the intestine could affect the efficacy of single pH-dependent drug delivery systems [29], such a combined system was selected for delivering the drug or nanoparticles to the desired site. The adopted coating process operative conditions, coating level and coating material compositions that were used in this study were previously set up by our team.

### 3.3. Release Studies

#### 3.3.1. Release of Budesonide from BCN and Uncoated BCNP

**Figure 4:**
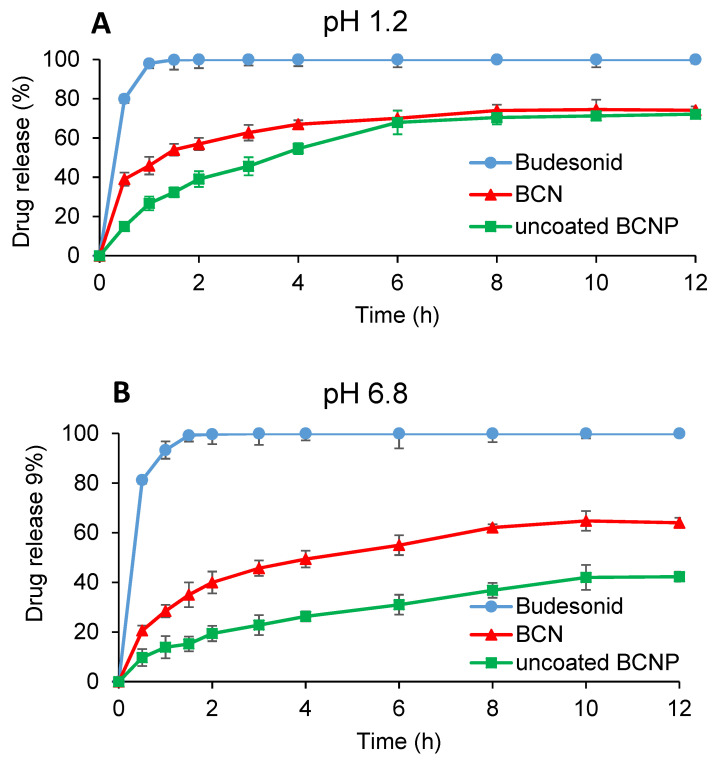
The drug release profile of budesonide, BCN and uncoated BCNP in (**A**) pH 1.2 and (**B**) pH 6.8.

Figure 4 shows the release profiles of BU powder, budesonide-loaded nanoparticles (BCN) and uncoated BCNP in simulated gastric fluid (SGF, pH 1.2) and simulated colonic fluid (SCF, pH 6.8). It could be observed that the loading of BU in chitosan nanoparticles resulted in a decline in the drug release rate. Similar findings were observed in other studies when drugs were encapsulated in polymeric matrices [62,68]. According to the results, the release profiles of BCN were pH-dependent which is not expected as BU solubility is not pH-dependent [58] and also it dissolves completely within the first 2 h at SGF and SCF. The unexpected pH-dependency of BCN formulations could be described by the properties of chitosan which is soluble in an acidic medium and conversely, but it has poor solubility in neutral pH [87], resulting in a faster release at pH 1.2 compared with pH 6.8. Release profiles of BCN in both pH levels presented a burst release of about 45% and 30% in pH 1.2 and 6.8, respectively during the first hour, followed by a sustained release behavior for up to 12 h. Initial burst release might be due to the dissolution of the drug which is located at or close to the nanoparticle surface which is more accessible to the release medium, and the hydrophilic nature of chitosan [88]. It can be seen that after 12 h, only 74% and 67% of the encapsulated drug were released from BCN in pH 1.2 and pH 6.8, respectively. The incomplete drug release from nanoparticles could be explained by the drug release mechanism from the chitosan matrix. Upon the exposure of chitosan to the aqueous media, a gel layer is formed around the drug particles [62]. Slow drug diffusion through this high viscose gel layer and chitosan matrix makes the dissolution profile sustained and incomplete. A slower release profile from pelletized nanoparticles compared with the BCN demonstrated that nanoparticles were not adversely affected by mechanical stresses during the extrusion and spheronization process.

#### 3.3.2. Release of Budesonide from Coated BCNP

**Figure 5:**
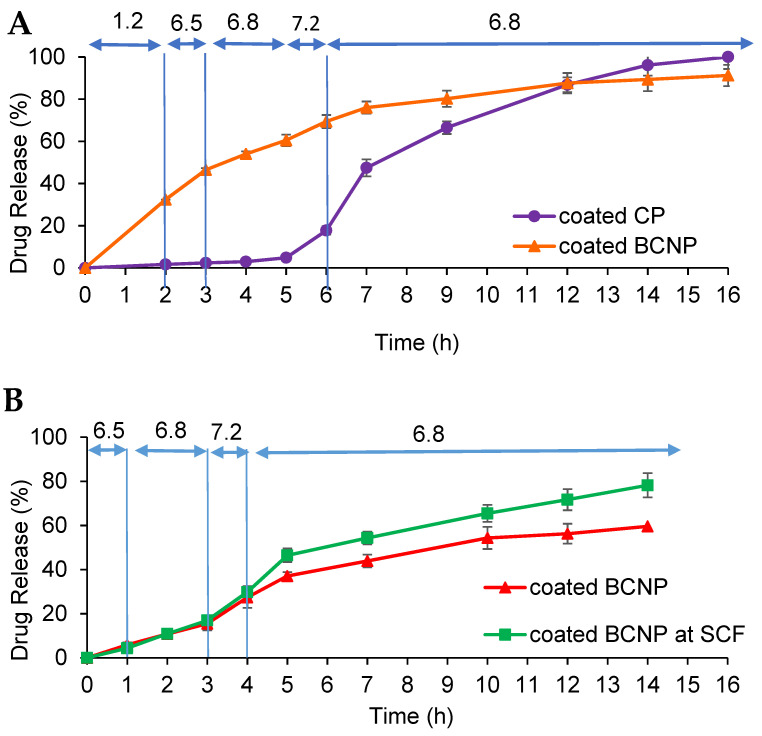
The drug release profile of coated pellets (**A**) in the continuous mode of dissolution test and (**B**) in the continuous mode of dissolution test except for the initial 2 h in pH 1.2.

As it can be seen in Figure 4, uncoated BCNP released more than 50% of the loaded drug during the first 2 h in acidic pH. Thus, to avoid early drug release and deliver most of the drug into the colon, pellets were coated with a combined pH and time-dependent coating system. In this case, continuous dissolution testing was performed in a pH progression medium for 16 h for coated CP and BCNP. The coated pellets exhibited a pH-dependent release profile (Figure 5A) as expected. No drug release was observed when coated conventional pellets passed through media with pH 1.2, 6.5, and 6.8 for 2, 1, and 2 h residence times, respectively. It was observed that while coated CP released almost 90% of the drug in the medium simulating the pH of the colonic environment, in the case of pellets containing nanoparticles such a coating system was less effective for delivering most of the drug to the colon. Coated BCNP released almost 30% and 35% of the drug in simulating pH of gastric and small intestine fluid, respectively; thus, only 35% of the drug was released in the final part of the continuous dissolution test. The lower efficiency of this coating system in the colon delivery of pellets containing nanoparticles can be attributed to the swelling properties of chitosan [89] and the composition of the coating. Although the coating composition is insoluble in the acidic medium it is permeable to it due to the presence of eudragit RS. As the acidic medium penetrates the pellets, chitosan would be swelled and could damage the functional coating. The swelling ratio of chitosan at low pH values is higher than neutral pH due to repulsion between similarly charged groups [90]. It seems that by preventing the entry of the acidic medium into the pellet, it is possible to prevent the destruction of the coating so a larger amount of drug could be delivered to the colon. To prove this hypothesis, the same continuous dissolution test except for the initial 2 h in pH 1.2 was performed with and without 4% *w*/*v* rat cecal content in the last medium with pH 6.8. As illustrated in Figure 5B, when the formulation was directly placed in the medium with pH 6.5 (bypassing the acidic environment) only 30% of the drug was released before reaching the colon-simulated media. Although it seems that the drug release from coated BCNP in such conditions might be incomplete (60% during 14 h), the presence of rat cecal content in the SCF medium increased the drug dissolution (80% during 14 h). This confirms the effect of the bacteria in the rat cecal as well as the pH sensitivity of the system to the drug release. This suggests that the degradation of chitosan in the pellets’ matrix might happen upon the action of the bacterial enzymes in the SCF [91], resulting in an increase in the release rate. The overall results indicated that in the case of coated BCNP, where the target of drug delivery is the colon, drug release in the stomach would be considered as a drawback [92], so more strategies such as using enteric-coated capsule [93] filled with the coated BCNP should be considered for oral delivery of this type of formulation where the target site is the colon area. However, when drug delivery is aimed at the entire GIT (Crohn’s disease) coated BCNP could provide a continuous release throughout the GIT. 

### 3.4. Morphological Characteristics of Coated BCNP

**Figure 6:**
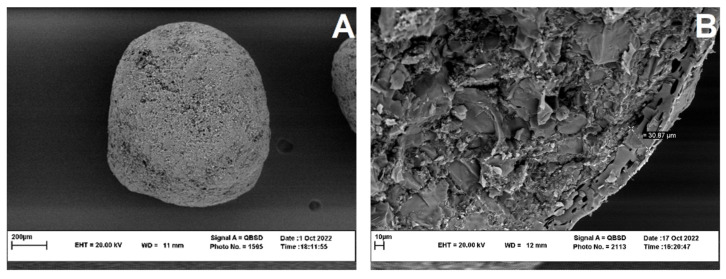
(**A**) Scanning electron microscopy of the coated BCNP and (**B**) the cross-sectional image of the coated BCNP.

Figure 6 illustrated the SEM images of the coated BCNP. These pellets were spherical and had smooth surfaces (Figure 6A). The cross-sectional image showed a uniform coat with approximately 30 µm thickness (Figure 6B).

### 3.5. In Vivo Therapeutic Efficacy in Rats

The therapeutic effects of the selected formulations were characterized by determining some indexes of colonic tissue edema (colitis activity index (CAI), colon/body weight, weight/length ratio of colon, the level of MDA and amount of GSH in colon tissue) [94], as well as histological studies. 

**Figure 7:**
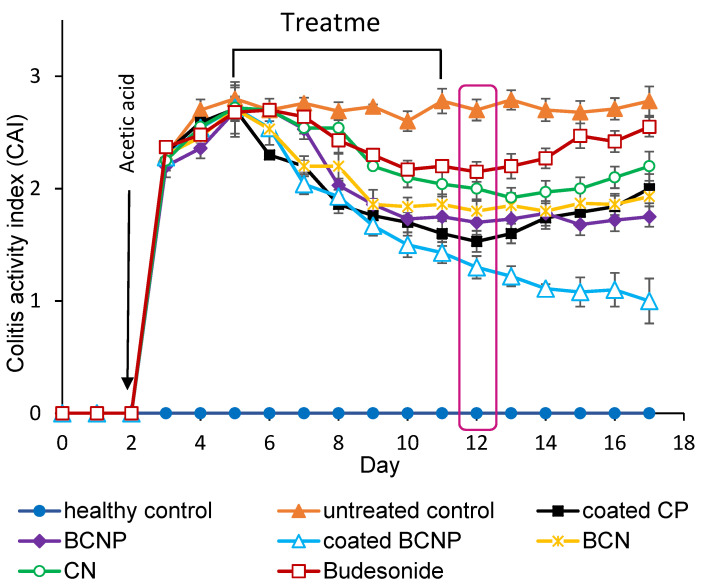
Colitis activity index for different rat groups.

The CAI is a clinical tool for the evaluation of the severity of inflammation in living animals [14]. After inducing experimental colitis, CAI increased rapidly (Figure 7); all animals showed loss of weight and diarrhea over the next 3 days indicating that all animals presented with colitis. All treatment groups showed a consistent decrease in CAI throughout the entire treatment period (days 5 to 11). On the day of sacrifice, all treated rats showed statistically lower CAI compared with the untreated control group (*p* < 0.05) in response to the decrease in intestinal inflammation [95]. While the group treated with free BU showed little improvement in the CAI, the coated BCNP-treated group demonstrated the most prominent decline in this index (two-fold reductions compared with the untreated group). For those rats which were not sacrificed on day 12, during the next 5 days of monitoring with no treatment (days 13 to 17), the increase in the inflammation severity was observed for both the free BU and coated CP treated groups, so that the CAI value significantly increased from day 12 to day 17, while the difference between CAI in this period was not significant for BCN, BCNP and CN treated groups. In the case of coated BCNP-treated rats, even though the administration of the drug was discontinued, surprisingly the healing process continued (Figure 7). A similar finding has been reported in another study in which the nanoparticle-treated rats continued to heal after stopping the treatment [96].

**Figure 8:**
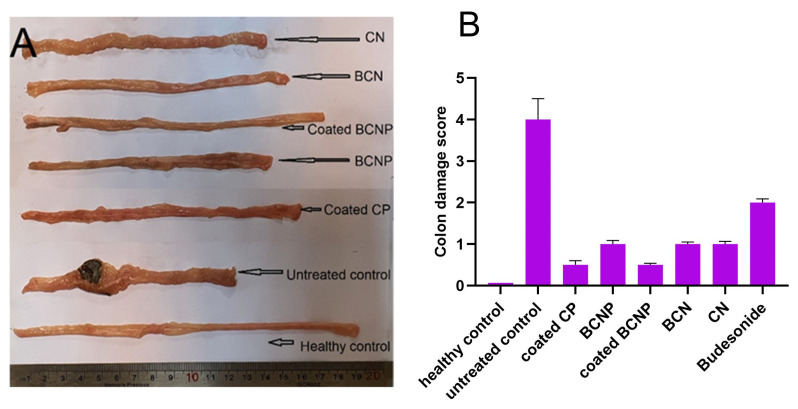
(**A**) Photographs for macroscopically examination and (**B**) colon damage score of the colon specimens resected from rats 24 h after the last drug administration.

As can be seen in Figure 8A, healthy rats had a colon length of 19 cm, while the colon length significantly decreased in untreated rats with values of ~11 cm. Additionally, untreated colitis rats showed signs of localized inflammation and ulceration and thickening of the colon wall. Although the conditions of the rats did not return to normal during the experiment period, it seems that the inflammatory signs were decreased in all of the treated groups when compared with the untreated group (Figure 8A). Based on the visual comparison, the most increase in colon length was observed in coated BCNP-treated group (almost 9 cm), and the colon length of this group was much close to the healthy group.

**Figure 9:**
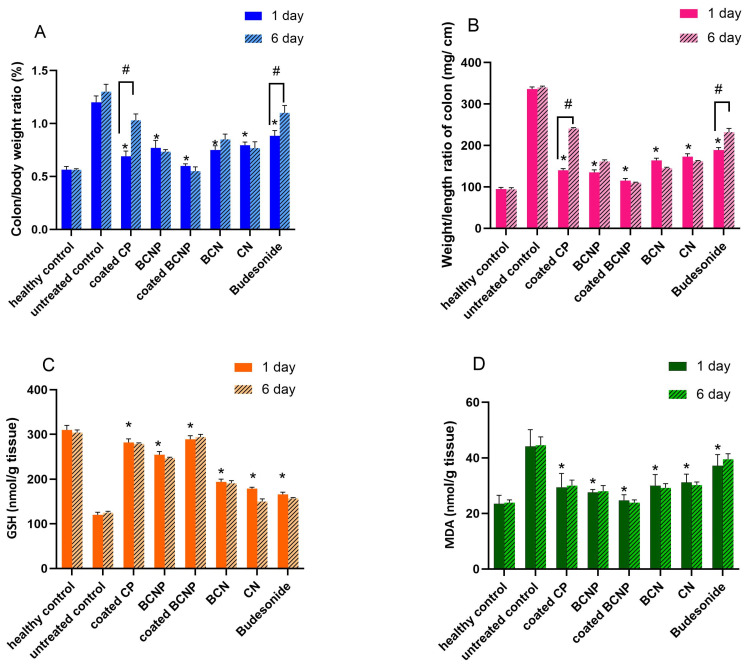
(**A**) Colon/body weight ratio; (**B**) Weight/length ratio of colon; (**C**) GSH and (**D**) level of MDA; determined 1 day or 6 days after final drug administration. * *p* < 0.05 comparison of treatment groups with untreated group after “1 day”. # *p* < 0.05 comparison of each group “1 day” and “6 day” after treatment.

As shown in Figure 9A,B, the colon/body weight ratio and weight/length ratio of the colon declined significantly in all of the treated groups when compared with the untreated control group (*p* < 0.05). These two indexes usually increase in response to the severity of inflammation [96]; therefore, the obtained results could indicate that all of the prescribed formulations had some anti-inflammatory effects. Regarding these two inflammatory indexes data, again, the most effective formulation for the treatment of inflammation was coated BCNP whilst free BU was less effective than others. Similar to the results obtained for CAI, BU and coated CP-treated groups showed a statistical difference when these two items (the colon/body weight ratio and weight/length ratio) were compared 6 days after the last drug administration (*p* < 0.05) while no significant differences for these items were observed in groups treated with nanoparticle-based formulations.

It is well-known that the amount of GSH is an indication of the anti-oxidative potential of the cells upon treatment and MDA is a marker indicating tissue injury [56]. A significant increase in the amount of GSH (Figure 9C), as well as a significant decrease in the level of MDA (Figure 9D), was observed in all treated groups compared with the untreated group. These results demonstrated that inflammation significantly decreased upon treatment of rats.

**Figure 10:**
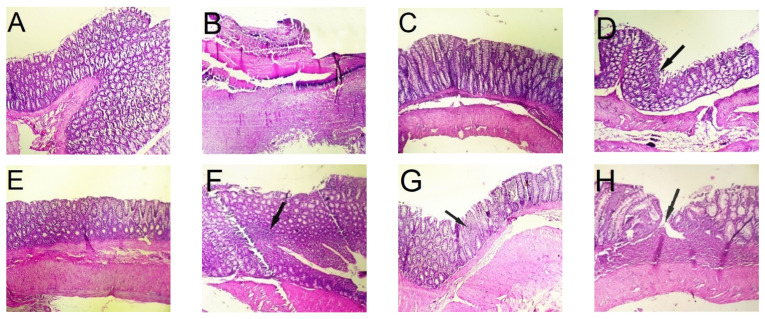
Histopathological characteristics of the colon tissues of the rats subjected to different treatments 24 h after the last drug administration. (**A**) Rat with normal colonic tissues; (**B**) untreated rat; (**C**) rat treated with coated CP; (**D**) rat treated with BCNP; (**E**) rat treated with coated BCNP; (**F**) rat treated with BCN; (**G**) rat treated with CN and (**H**) rat treated with Budesonide. (arrows point to area of inflammation).

Figure 10 shows the histopathological characteristics of the colon’s tissues and Figure 8B presents a colitis damage score. Light microscopic observation of the colon tissue of healthy rats demonstrated normal colon histology with no signs of unusual tissue architecture or abnormal tissue morphology (Figure 10A) which was scored as zero for colitis. By contrast, in untreated colitis rats the utmost tissue damage, and goblet cell depletion associated with disruption and necrotic mucosal structure was seen (Figure 10B) which corresponds to the highest score (four) of colitis. Although the histological sections of the colonic tissues in all treatment groups showed some signs of improvement compared with the untreated group (Figure 10C–H), in the colon tissue of free budesonide-treated groups signs of smooth muscle thickening were seen (Figure 10H) which was scored as two for colitis. A much closer condition to the normal colon histology was seen in rats treated with coated BCNP and coated CP (Figure 10C,E). Observation of colon tissue of other treated groups (BCNP, BCN and CN) demonstrated normal colon histology; however, trace regions of focal inflammatory cell infiltration were seen (Figure 10D,F,G) which was scored one for colitis.

Overall, results from the in vivo studies demonstrated that all prescribed formulations significantly are effective in colitis treatment. A decrease in inflammation in CN-treated rats could be related to the anti-inflammatory effects of chitosan in IBD [59]. 

Formulation containing nanoparticles was found to be more effective than free BU formulation. This could be due to the absorption of a higher amount of free drug in the upper sites of GIT which resulted in inadequate amounts of drug delivered to the inflamed colon [97]. The high efficacy of nanoparticles in the treatment of colitis indicates the presence of higher amounts of the drug at the site of inflammation, confirming the greater accumulation of small-sized particles at the site of inflammation [13,98] and the ease of their adherence to the thick mucus layer [99]. In addition, polymeric nanoparticles would protect drug release in the upper site of GIT and allow for a prolonged residence at the surface of the release site [68]. 

In this study, nanoparticles were turned into pellet dosage form to increase the clinical effects by reducing the probability of premature uptake in the small intestine. Although histological characteristics of BCN and BCNP-treated rats demonstrated similar therapeutic effects, according to the other in vivo results the superiority of BCNP is obvious. Degradation of free nanoparticles in oral delivery and during their passage in upper parts of the GIT could result in a lower drug amount delivered to the colon [6] while incorporating the nanoparticles in the matrix of pellets could improve local stability within the gastrointestinal tract [100].

During the treatment period, coated BCNP formulation showed the most anti-inflammatory effects; however, regarding the amount of GSH, level of MDA and histological assessment of colon tissues, significant differences with the coated CP were not clearly visible. The superiority of coated BCNP formulation in in vivo studies in rats could be related to the shorter and faster movement of delivery systems in the intestine in rats compared with humans [101], which could take more drugs to the site of action. 

The higher efficacy of coated systems (coated BCNP and coated CP) compared with others is due to their coating system which allows the maximum delivery of the drug to the target site without early release in the stomach or upper sections of the gastrointestinal tract after taking the formulation orally. 

Although it seems that with a proper coating system colon targeting of BU pellets could effectively be achieved, the advantages of using pelletized BU nanoparticles would be bolded after stopping drug administration. After treatment discontinuation, the group receiving coated CP showed a fast relapse, whereas this was not the case in rats treated with coated BCNP. Similar findings previously had been observed for PLGA nanoparticles [96,98]. These differences may suggest a higher drug accumulation in the inflamed tissue after treatment with nanoparticles and remaining drug carriers in the inflamed regions for up to several days. This claim could be confirmed by an examination of blood glucose levels.

### 3.6. Blood Glucose Level

**Figure 11:**
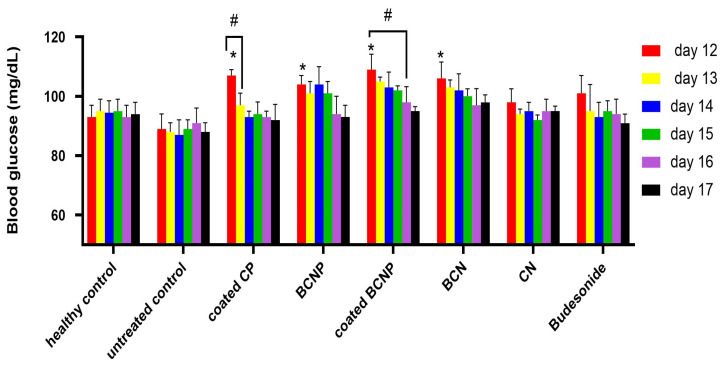
Blood glucose level (mg/dL) in animals on 12th day to 17th day of study. * *p* < 0.05 comparison of treatment groups with untreated group. # *p* < 0.05 comparison of each group on different days.

Glucocorticoids are known to increase blood glucose levels due to their potential for enhancing intestinal sugar uptake [58]. Thus, for determination of the potency of formulations which their aim is local drug delivery to the intestinal mucosa and accumulation in it, the level of blood glucose of the animals could be considered as an indicator. As it can be seen in Figure 11, the blood glucose level of all rats treated with a formulation containing the drug increased progressively compared with untreated rats. These results indicate that a certain amount of drug was available in the inflamed region on the test day. However, glucose levels were significantly higher in the group of rats treated with nanoparticles containing BCN and BCNP compared with the groups treated with free drugs. The increased glucose level could suggest more extent of targeted drug delivery to the site of inflammation by using nanotechnology. Similar results had been seen previously in other studies [48,57]. The blood glucose level was significantly higher in the rats treated with coated CP and coated BCNP compared with the other treated groups (110 and 109 mg/dl, respectively), which is related to the higher amounts of drug that were delivered to the colon by avoiding release in the upper parts of GIT or premature uptake of the drug due to the coating system. However, it was observed that animals treated with coated CP revealed a remarkable decrease in blood glucose concentration within 24 h after the first examination, while in animals treated with coated BCNP, this reduction become significant after four days. These results also confirm higher drug accumulation after treatment with nanoparticles in comparison with other groups which may last for up to several days and are in agreement with other in vivo results.

## 4. Conclusions

To overcome the limitations of oral delivery of budesonide nanoparticles in colon delivery, a novel formulation based on functional coating of pelletized nanoparticles was proposed. Although in vitro dissolution test results did not indicate the superiority of this formulation over conventional coated pellets (due to the effect of acidic conditions on chitosan), in vivo results demonstrated the supplementary effect of nanoformulation of budesonide and the encapsulation in a properly coated pellet system for colon delivery that resulted in a synergistic and long-lasting anti-inflammatory effect in comparison with coated pellets containing free drug. 

## Figures and Tables

**Table 1:** Size, PDI, zeta potential and EE for different nanoparticles.

Formulation	Size (nm)	PDI	Zeta (mV)	EE (%)
BCN-1	288 ± 7.5	0.33 ± 0.02	+26.6 ± 1.1	37.2 ± 3.1
BCN-2	323 ± 5.1	0.43 ± 0.05	+27.9 ± 0.9	29.1 ± 2.4
BCN-3	416 ± 6.8	0.44 ± 0.03	+30.6 ± 1.4	10.8 ± 1.1
BCN-4	566 ± 7.7	0.45 ± 0.01	+31.0 ± 1.2	2.2 ± 0.9

## Data Availability

Not applicable.

## References

[1] Hua S., Marks E., Schneider J.J., Keely S. (2015). Advances in oral nano-delivery systems for colon targeted drug delivery in inflammatory bowel disease: Selective targeting to diseased versus healthy tissue. Nanomed. Nanotechnol. Biol. Med..

[2] Yan Y., Ren F., Wang P., Sun Y., Xing J. (2019). Synthesis and evaluation of a prodrug of 5-aminosalicylic acid for the treatment of ulcerative colitis. Iran. J. Basic Med. Sci..

[3] Chen M., Yu Y., Yang S., Yang D. (2021). Pretreatment with licochalcone a enhances therapeutic activity of rat bone marrow mesenchymal stem cells in animal models of colitis. Iran. J. Basic Med. Sci..

[4] Ali H., Weigmann B., Neurath M., Collnot E., Windbergs M., Lehr C.-M. (2014). Budesonide loaded nanoparticles with pH-sensitive coating for improved mucosal targeting in mouse models of inflammatory bowel diseases. J. Control. Release.

[5] Lahiff C., Kane S., Moss A.C. (2011). Drug development in inflammatory bowel disease: The role of the FDA. Inflamm. Bowel Dis..

[6] Meißner Y., Pellequer Y., Lamprecht A. (2006). Nanoparticles in inflammatory bowel disease: Particle targeting versus pH-sensitive delivery. Int. J. Pharm..

[7] Xiao B., Laroui H., Ayyadurai S., Viennois E., Charania M.A., Zhang Y., Merlin D. (2013). Mannosylated bioreducible nanoparticle-mediated macrophage-specific TNF-α RNA interference for IBD therapy. Biomaterials.

[8] Ding S., Zhang N., Lyu Z., Zhu W., Chang Y.-C., Hu X., Du D., Lin Y. (2021). Protein-based nanomaterials and nanosystems for biomedical applications: A review. Mater. Today.

[9] Li D.-F., Yang M.-F., Xu H.-M., Zhu M.-Z., Zhang Y., Tian C.-M., Nie Y.-Q., Wang J.-Y., Liang Y.-J., Yao J. (2022). Nanoparticles for oral delivery: Targeted therapy for inflammatory bowel disease. J. Mater. Chem. B.

[10] Press A.G., A Hauptmann I., Hauptmann L., Fuchs B., Ewe K., Fuchs M., Ramadori G., Ewe K. (1998). Gastrointestinal pH profiles in patients with inflammatory bowel disease. Aliment. Pharmacol. Ther..

[11] Desai M.P., Labhasetwar V., Amidon G.L., Levy R.J. (1996). Gastrointestinal Uptake of Biodegradable Microparticles: Effect of Particle Size. Pharm. Res..

[12] Lamprecht A., Yamamoto H., Takeuchi H., Kawashima Y. (2005). Nanoparticles Enhance Therapeutic Efficiency by Selectively Increased Local Drug Dose in Experimental Colitis in Rats. J. Pharmacol. Exp. Ther..

[13] Brusini R., Varna M., Couvreur P. (2020). Advanced nanomedicines for the treatment of inflammatory diseases. Adv. Drug Deliv. Rev..

[14] Tang H., Xiang D., Wang F., Mao J., Tan X., Wang Y. (2017). 5-ASA-loaded SiO_2_ nanoparticles-a novel drug delivery system targeting therapy on ulcerative colitis in mice. Mol. Med. Rep..

[15] Ali H., Weigmann B., Collnot E.-M., Khan S.A., Windbergs M., Lehr C.-M. (2015). Budesonide Loaded PLGA Nanoparticles for Targeting the Inflamed Intestinal Mucosa—Pharmaceutical Characterization and Fluorescence Imaging. Pharm. Res..

[16] Vafaei S.Y., Esmaeili M., Amini M., Atyabi F., Ostad S.N., Dinarvand R. (2016). Self assembled hyaluronic acid nanoparticles as a potential carrier for targeting the inflamed intestinal mucosa. Carbohydr. Polym..

[17] Beloqui A., Coco R., Alhouayek M., Solinís M., Rodríguez-Gascón A., Muccioli G.G., Préat V. (2013). Budesonide-loaded nanostructured lipid carriers reduce inflammation in murine DSS-induced colitis. Int. J. Pharm..

[18] Han H.-K., Shin H.-J., Ha D.H. (2012). Improved oral bioavailability of alendronate via the mucoadhesive liposomal delivery system. Eur. J. Pharm. Sci..

[19] Cu Y., Saltzman W.M. (2009). Controlled Surface Modification with Poly(ethylene)glycol Enhances Diffusion of PLGA Nanoparticles in Human Cervical Mucus. Mol. Pharm..

[20] Barea M.J., Jenkins M.J., Lee Y.S., Johnson P., Bridson R.H. (2012). Encapsulation of Liposomes within pH Responsive Microspheres for Oral Colonic Drug Delivery. Int. J. Biomater..

[21] Laroui H., Dalmasso G., Nguyen H.T.T., Yan Y., Sitaraman S.V., Merlin D. (2010). Drug-Loaded Nanoparticles Targeted to the Colon With Polysaccharide Hydrogel Reduce Colitis in a Mouse Model. Gastroenterology.

[22] Zhang M., Merlin D. (2018). Nanoparticle-Based Oral Drug Delivery Systems Targeting the Colon for Treatment of Ulcerative Colitis. Inflamm. Bowel Dis..

[23] Wilson D.S., Dalmasso G., Wang L., Sitaraman S.V., Merlin D., Murthy N. (2010). Orally delivered thioketal nanoparticles loaded with TNF-α–siRNA target inflammation and inhibit gene expression in the intestines. Nat. Mater..

[24] Ashford M., Fell J.T., Attwood D., Sharma H., Woodhead P.J. (1993). An in vivo investigation into the suitability of pH dependent polymers for colonic targeting. Int. J. Pharm..

[25] Wang N., Shao L., Lu W., Fang W., Zhang Q., Sun L., Gao S., Zhu Q., Chen S., Hu R. (2022). 5-aminosalicylic acid pH sensitive core-shell nanoparticles targeting ulcerative colitis. J. Drug Deliv. Sci. Technol..

[26] Cai X., Wang X., He M., Wang Y., Lan M., Zhao Y., Gao F. (2021). Colon-targeted delivery of tacrolimus using pH-responsive polymeric nanoparticles for murine colitis therapy. Int. J. Pharm..

[27] Zhou H., Qian H. (2018). Preparation and characterization of pH-sensitive nanoparticles of budesonide for the treatment of ulcerative colitis. Drug Des. Dev. Ther..

[28] Turanlı Y., Acartürk F. (2021). Fabrication and characterization of budesonide loaded colon-specific nanofiber drug delivery systems using anionic and cationic polymethacrylate polymers. J. Drug Deliv. Sci. Technol..

[29] Naeem M., Choi M., Cao J., Lee Y., Ikram M., Yoon S., Lee J., Moon H.R., Kim M., Jung Y. (2015). Colon-targeted delivery of budesonide using dual pH- and time-dependent polymeric nanoparticles for colitis therapy. Drug Des. Dev. Ther..

[30] Sinhmar G.K., Shah N.N., Chokshi N.V., Khatri H.N., Patel M.M. (2018). Process, optimization, and characterization of budesonide-loaded nanostructured lipid carriers for the treatment of inflammatory bowel disease. Drug Dev. Ind. Pharm..

[31] Lamprecht A., Yamamoto H., Takeuchi H., Kawashima Y. (2005). A pH-sensitive microsphere system for the colon delivery of tacrolimus containing nanoparticles. J. Control. Release.

[32] Rodriguez M., Antúnez J.A., Taboada C., Seijo B., Torres D. (2001). Colon-specific delivery of budesonide from microencapsulated cellulosic cores: Evaluation of the efficacy against colonic inflammation in rats. J. Pharm. Pharmacol..

[33] Krishnamachari Y., Madan P., Lin S. (2007). Development of pH- and time-dependent oral microparticles to optimize budesonide delivery to ileum and colon. Int. J. Pharm..

[34] Patel M.M., Amin A.F. (2011). Process, optimization and characterization of mebeverine hydrochloride loaded guar gum microspheres for irritable bowel syndrome. Carbohydr. Polym..

[35] Barea M., Jenkins M., Gaber M., Bridson R. (2010). Evaluation of liposomes coated with a pH responsive polymer. Int. J. Pharm..

[36] Leonard F., Ali H., Collnot E.-M., Crielaard B.J., Lammers T., Storm G. (2012). Screening of budesonide nanoformulations for treatment of inflammatory bowel disease in an inflamed 3D cell-culture model. Altex—Altern. Anim. Exp..

[37] Smitha K., Anitha A., Furuike T., Tamura H., Nair S.V., Jayakumar R. (2013). In vitro evaluation of paclitaxel loaded amorphous chitin nanoparticles for colon cancer drug delivery. Colloids Surf. B.

[38] Chen M.-C., Mi F.-L., Liao Z.-X., Hsiao C.-W., Sonaje K., Chung M.-F., Hsu L.-W., Sung H.-W. (2013). Recent advances in chitosan-based nanoparticles for oral delivery of macromolecules. Adv. Drug Deliv. Rev..

[39] Ahmed O., Abdel-Halim M., Farid A., Elamir A. (2022). Taurine loaded chitosan-pectin nanoparticle shows curative effect against acetic acid-induced colitis in rats. Chem. Interact..

[40] Coco R., Plapied L., Pourcelle V., Jérôme C., Brayden D.J., Schneider Y.-J., Préat V. (2013). Drug delivery to inflamed colon by nanoparticles: Comparison of different strategies. Int. J. Pharm..

[41] Michailidou G., Ainali N.M., Xanthopoulou E., Nanaki S., Kostoglou M., Koukaras E.N., Bikiaris D.N. (2020). Effect of Poly(vinyl alcohol) on Nanoencapsulation of Budesonide in Chitosan Nanoparticles via Ionic Gelation and Its Improved Bioavailability. Polymers.

[42] de Pinho Neves A.L., Milioli C.C., Müller L., Riella H.G., Kuhnen N.C., Stulzer H.K. (2014). Factorial design as tool in chitosan nanoparticles development by ionic gelation technique. Colloids Surf. A Physicochem. Eng. Asp..

[43] Koukaras E., Papadimitriou S.A., Bikiaris D.N., Froudakis G.E. (2012). Insight on the Formation of Chitosan Nanoparticles through Ionotropic Gelation with Tripolyphosphate. Mol. Pharm..

[44] Soltani F., Kamali H., Akhgari A., Garekani H.A., Nokhodchi A., Sadeghi F. (2022). Different trends for preparation of budesonide pellets with enhanced dissolution rate. Adv. Powder Technol..

[45] Sardou H.S., Akhgari A., Mohammadpour A.H., Namdar A.B., Kamali H., Jafarian A.H., Garekani H.A., Sadeghi F. (2022). Optimization study of combined enteric and time-dependent polymethacrylates as a coating for colon targeted delivery of 5-ASA pellets in rats with ulcerative colitis. Eur. J. Pharm. Sci..

[46] de Assis P.O.A., Guerra G.C.B., de Souza Araújo D.F., de Araújo Júnior R.F., Machado T.A.D.G., de Araújo A.A., de Lima T.A.S., Garcia H.E.M., de Fátima Leal Interaminense de Andrade L., de Cássia Ramos do Egypto Queiroga R. (2016). Intestinal anti-inflammatory activity of goat milk and goat yoghurt in the acetic acid model of rat colitis. Int. Dairy J..

[47] Guerra G.C., Araújo A., Lira G.A., Melo M.N., Souto K.K., Fernandes D., Silva A.L., Araújo R.F. (2015). Telmisartan decreases inflammation by modulating TNF-α, IL-10, and RANK/RANKL in a rat model of ulcerative colitis. Pharmacol. Rep..

[48] Qelliny M.R., Aly U.F., Elgarhy O.H., Khaled K.A. (2019). Budesonide-Loaded Eudragit S 100 Nanocapsules for the Treatment of Acetic Acid-Induced Colitis in Animal Model. AAPS PharmSciTech.

[49] Sardou H.S., Akhgari A., Mohammadpour A.H., Kamali H., Jafarian A.H., Garekani H.A., Sadeghi F. (2021). Application of inulin/Eudragit RS in 5-ASA pellet coating with tuned, sustained-release feature in an animal model of ulcerative colitis. Int. J. Pharm..

[50] Ferri D., Costero A.M., Gaviña P., Parra M., Merino V., Teruel A.H., Sancenón F., Martínez-Máñez R. (2019). Efficacy of budesonide-loaded mesoporous silica microparticles capped with a bulky azo derivative in rats with TNBS-induced colitis. Int. J. Pharm..

[51] Dai C., Zheng C.-Q., Meng F.-J., Zhou Z., Sang L.-X., Jiang M. (2012). VSL#3 probiotics exerts the anti-inflammatory activity via PI3k/Akt and NF-κB pathway in rat model of DSS-induced colitis. Mol. Cell. Biochem..

[52] Alhouayek M., Lambert D.M., Delzenne N.M., Cani P.D., Muccioli G.G. (2011). Increasing endogenous 2-arachidonoylglycerol levels counteracts colitis and related systemic inflammation. FASEB J..

[53] Bayat A., Dorkoosh F.A., Dehpour A.R., Moezi L., Larijani B., Junginger H.E., Rafiee-Tehrani M. (2008). Nanoparticles of quaternized chitosan derivatives as a carrier for colon delivery of insulin: Ex vivo and in vivo studies. Int. J. Pharm..

[54] Gareb B., Dijkstra G., Kosterink J.G., Frijlink H.W. (2018). Development of novel zero-order release budesonide tablets for the treatment of ileo-colonic inflammatory bowel disease and comparison with formulations currently used in clinical practice. Int. J. Pharm..

[55] Uchiyama M., Mihara M. (1978). Determination of malonaldehyde precursor in tissues by thiobarbituric acid test. Anal. Biochem..

[56] Mehri S., Meshki M.A., Hosseinzadeh H. (2015). Linalool as a neuroprotective agent against acrylamide-induced neurotoxicity in Wistar rats. Drug Chem. Toxicol..

[57] Yoo J.-W., Naeem M., Cao J., Choi M., Kim W., Moon H.R., Lee B.L., Kim M.-S., Jung Y. (2015). Enhanced therapeutic efficacy of budesonide in experimental colitis with enzyme/pH dual-sensitive polymeric nanoparticles. Int. J. Nanomed..

[58] Thiesen A., E Wild G., A Tappenden K., Drozdowski L., Keelan M., A Thomson B.K., I McBurney M., Clandinin M.T., Thomson A.B.R. (2003). The locally acting glucocorticosteroid budesonide enhances intestinal sugar uptake following intestinal resection in rats. Gut.

[59] Ribeiro J.C.V., Forte T.C.M., Tavares S.J.S., Andrade F.K., Vieira R.S., Lima V. (2021). The effects of the molecular weight of chitosan on the tissue inflammatory response. J. Biomed. Mater. Res. Part A.

[60] Niu W., Dong Y., Fu Z., Lv J., Wang L., Zhang Z., Huo J., Ju J. (2021). Effects of molecular weight of chitosan on anti-inflammatory activity and modulation of intestinal microflora in an ulcerative colitis model. Int. J. Biol. Macromol..

[61] Xu Y., Du Y. (2002). Effect of molecular structure of chitosan on protein delivery properties of chitosan nanoparticles. Int. J. Pharm..

[62] Yang H.-C., Hon M.-H. (2009). The effect of the molecular weight of chitosan nanoparticles and its application on drug delivery. Microchem. J..

[63] Bhatt H., Naik B., Dharamsi A. (2014). Solubility Enhancement of Budesonide and Statistical Optimization of Coating Variables for Targeted Drug Delivery. J. Pharm..

[64] Kouchak M., Avadi M., Abbaspour M., Jahangiri A., Boldaji S.K. (2012). Effect of different molecular weights of chitosan on preparation and characterization of insulin loaded nanoparticles by ion gelation method. Int. J. Drug Dev. Res..

[65] Desai K.G. (2016). Chitosan Nanoparticles Prepared by Ionotropic Gelation: An Overview of Recent Advances. Crit. Rev. Ther. Drug Carr. Syst..

[66] Schmidt C., Lautenschlaeger C., Collnot E.-M., Schumann M., Bojarski C., Schulzke J.-D., Lehr C.-M., Stallmach A. (2013). Nano- and microscaled particles for drug targeting to inflamed intestinal mucosa—A first in vivo study in human patients. J. Control. Release.

[67] Dube A., Nicolazzo J.A., Larson I. (2010). Chitosan nanoparticles enhance the intestinal absorption of the green tea catechins (+)-catechin and (−)-epigallocatechin gallate. Eur. J. Pharm. Sci..

[68] Elzatahry A.A., Eldin M.M. (2008). Preparation and characterization of metronidazole-loaded chitosan nanoparticles for drug delivery application. Polym. Adv. Technol..

[69] Fan W., Yan W., Xu Z., Ni H. (2012). Formation mechanism of monodisperse, low molecular weight chitosan nanoparticles by ionic gelation technique. Colloids Surf. B Biointerfaces.

[70] Gomathi T., Sudha P., Florence J.A.K., Venkatesan J., Anil S. (2017). Fabrication of letrozole formulation using chitosan nanoparticles through ionic gelation method. Int. J. Biol. Macromol..

[71] Joseph J.J., Sangeetha D., Gomathi T. (2016). Sunitinib loaded chitosan nanoparticles formulation and its evaluation. Int. J. Biol. Macromol..

[72] Bouwman A.M., Heijstra M.P., Schaefer N.C., Duiverman E.J., Lesouëf P.N., Devadason S.G. (2006). Improved Efficiency of Budesonide Nebulization Using Surface-Active Agents. Drug Deliv..

[73] Abouelhag H., Sivakumar S., Bagul U., Eltyep E.M., Safhi M. (2017). Preparation and physical characterization of cisplatin chitosan nanoparticles by zeta nano sizer “prime step for formulation and development”. Int. J. Pharm. Sci. Res..

[74] Souza T.G.F., Ciminelli V.S.T., Mohallem N.D.S. (2016). A comparison of TEM and DLS methods to characterize size distribution of ceramic nanoparticles. J. Phys. Conf. Ser..

[75] Tuoriniemi J., Johnsson A.-C.J.H., Perez Holmberg J., Gustafsson S., Gallego-Urrea J.A., Olsson E., Pettersson J.B.C., Hassellöv M. (2014). Intermethod comparison of the particle size distributions of colloidal silica nanoparticles. Sci. Technol. Adv. Mater..

[76] Vafaei S.Y., Dinarvand R., Esmaeili M., Mahjub R., Toliyat T. (2015). Controlled-release drug delivery system based on fluocinolone acetonide–cyclodextrin inclusion complex incorporated in multivesicular liposomes. Pharm. Dev. Technol..

[77] Colby A.H., Liu R., Doyle R.P., Merting A., Zhang H., Savage N., Chu N.-Q., Hollister B.A., McCulloch W., Burdette J.E. (2021). Pilot-scale production of expansile nanoparticles: Practical methods for clinical scale-up. J. Control. Release.

[78] Kumar A., Sawant K. (2013). Encapsulation of exemestane in polycaprolactone nanoparticles: Optimization, characterization, and release kinetics. Cancer Nanotechnol..

[79] Ferrero F., Periolatto M. (2012). Antimicrobial finish of textiles by chitosan UV-curing. J. Nanosci. Nanotechnol..

[80] Ntohogian S., Gavriliadou V., Christodoulou E., Nanaki S., Lykidou S., Naidis P., Mischopoulou L., Barmpalexis P., Nikolaidis N., Bikiaris D.N. (2018). Chitosan Nanoparticles with Encapsulated Natural and UF-Purified Annatto and Saffron for the Preparation of UV Protective Cosmetic Emulsions. Molecules.

[81] Bruni G., Maggi L., Tammaro L., Canobbio A., Di Lorenzo R., D’Aniello S., Domenighini C., Berbenni V., Milanese C., Marini A. (2015). Fabrication, Physico-Chemical, and Pharmaceutical Characterization of Budesonide-Loaded Electrospun Fibers for Drug Targeting to the Colon. J. Pharm. Sci..

[82] Papadimitriou S., Bikiaris D., Avgoustakis K., Karavas E., Georgarakis M. (2008). Chitosan nanoparticles loaded with dorzolamide and pramipexole. Carbohydr. Polym..

[83] Aghrbi I., Fülöp V., Jakab G., Kállai-Szabó N., Balogh E., Antal I. (2021). Nanosuspension with improved saturated solubility and dissolution rate of cilostazol and effect of solidification on stability. J. Drug Deliv. Sci. Technol..

[84] Chopra R., Podczeck F., Newton J., Alderborn G. (2002). The influence of pellet shape and film coating on the filling of pellets into hard shell capsules. Eur. J. Pharm. Biopharm..

[85] Davis S.S., Hardy J.G., Fara J.W. (1986). Transit of pharmaceutical dosage forms through the small intestine. Gut.

[86] Kanwar N., Kumar R., Sinha V. (2015). Preparation and Evaluation of Multi-Particulate System (Pellets) of Prasugrel Hydrochloride. Open Pharm. Sci. J..

[87] Motwani S.K., Chopra S., Talegaonkar S., Kohli K., Ahmad F., Khar R.K. (2008). Chitosan–sodium alginate nanoparticles as submicroscopic reservoirs for ocular delivery: Formulation, optimisation and in vitro characterisation. Eur. J. Pharm. Biopharm..

[88] Egusquiaguirre S.P., Manguán-García C., Pintado-Berninches L., Iarriccio L., Carbajo D., Albericio F., Royo M., Pedraz J.L., Hernández R.M., Perona R. (2015). Development of surface modified biodegradable polymeric nanoparticles to deliver GSE24.2 peptide to cells: A promising approach for the treatment of defective telomerase disorders. Eur. J. Pharm. Biopharm..

[89] Huanbutta K., Cheewatanakornkool K., Terada K., Nunthanid J., Sriamornsak P. (2013). Impact of salt form and molecular weight of chitosan on swelling and drug release from chitosan matrix tablets. Carbohydr. Polym..

[90] Karaaslan M.A., Tshabalala M.A., Buschle-Diller G. (2010). Wood hemicellulose/chitosan-based semi-interpenetrating network hydrogels: Mechanical, swelling and controlled drug release properties. BioResources.

[91] Zhang H., Neau S.H. (2002). In vitro degradation of chitosan by bacterial enzymes from rat cecal and colonic contents. Biomaterials.

[92] Kobayashi T., Siegmund B., Le Berre C., Wei S.C., Ferrante M., Shen B., Bernstein C.N., Danese S., Peyrin-Biroulet L., Hibi T. (2020). Ulcerative colitis. Nat. Rev. Dis. Prim..

[93] Sonaje K., Chen Y.-J., Chen H.-L., Wey S.-P., Juang J.-H., Nguyen H.-N., Hsu C.-W., Lin K.-J., Sung H.-W. (2010). Enteric-coated capsules filled with freeze-dried chitosan/poly(γ-glutamic acid) nanoparticles for oral insulin delivery. Biomaterials.

[94] Mura C., Nácher A., Merino V., Merino-Sanjuan M., Carda C., Ruiz A., Manconi M., Loy G., Fadda A., Diez-Sales O. (2011). N-Succinyl-chitosan systems for 5-aminosalicylic acid colon delivery: In vivo study with TNBS-induced colitis model in rats. Int. J. Pharm..

[95] Walsh A.J., Bryant R.V., Travis S.P. (2016). Current best practice for disease activity assessment in IBD. Nat. Rev. Gastroenterol. Hepatol..

[96] Makhlof A., Tozuka Y., Takeuchi H. (2009). pH-Sensitive nanospheres for colon-specific drug delivery in experimentally induced colitis rat model. Eur. J. Pharm. Biopharm..

[97] Varshosaz J., Emami J., Fassihi A., Tavakoli N., Minaiyan M., Ahmadi F., Mahzouni P., Dorkoosh F. (2010). Effectiveness of budesonide-succinate-dextran conjugate as a novel prodrug of budesonide against acetic acid-induced colitis in rats. Int. J. Color. Dis..

[98] Lamprecht A., Ubrich N., Yamamoto H., Schäfer U., Takeuchi H., Maincent P., Kawashima Y., Lehr C.-M. (2001). Biodegradable nanoparticles for targeted drug delivery in treatment of inflammatory bowel disease. J. Pharmacol. Exp. Ther..

[99] Lamprecht A., Schäfer U., Lehr C.-M.M. (2001). Size-Dependent bioadhesion of micro- and nanoparticulate carriers to the inflamed colonic mucosa. Pharm. Res..

[100] Schmidt C., Bodmeier R. (1999). Incorporation of polymeric nanoparticles into solid dosage forms. J. Control. Release.

[101] McConnell E.L., Basit A.W., Murdan S. (2008). Measurements of rat and mouse gastrointestinal pH, fluid and lymphoid tissue, and implications for in-vivo experiments. J. Pharm. Pharmacol..

